# The L-type Voltage-Gated Calcium Channel co-localizes with Syntaxin 1A in nano-clusters at the plasma membrane

**DOI:** 10.1038/s41598-017-10588-4

**Published:** 2017-09-12

**Authors:** Julia Sajman, Michael Trus, Daphne Atlas, Eilon Sherman

**Affiliations:** 1 Racah Institute of Physics, The Hebrew University of Jerusalem, Jerusalem, 91904 Israel; 2 Dept. of Biological Chemistry, Institute of Life Sciences, Jerusalem, 91904 Israel

## Abstract

The secretory signal elicited by membrane depolarization traverses from the Ca^2+^-bound α_1_1.2 pore-forming subunit of the L-type Ca^2+^-channel (Cav1.2) to syntaxin 1 A (Sx1A) via an intra-membrane signaling mechanism. Here, we report the use of two-color Photo-Activated-Localization-Microscopy (PALM) to determine the relation between Cav1.2 and Sx1A in single-molecule detail. We observed nanoscale co-clusters of PAmCherry-tagged Sx1A and Dronpa-tagged α_1_1.2 at a ~1:1 ratio. PAmCherry-tagged Sx1A^C145A^, or PAmCherry-tagged Sx2, an inactive Cav1.2 modulator, in which Cys145 is a Ser residue, showed no co-clustering. These results are  consistent with the crucial role of the single cytosolic Sx1ACys145 in clustering with Cav1.2. Cav1.2 and the functionally inactive transmembrane-domain double mutant Sx1A^C271V/C272V^ engendered clusters with a ~2:1 ratio. A higher extent of co-clustering, which coincides with compromised depolarization-evoked transmitter-release, was observed also by oxidation of Sx1ACys271 and Cys272. Our super-resolution-imaging results set the stage for studying co-clustering of the channel with other exocytotic proteins at a single-molecule level.

## Introduction

Fast neurotransmitter release is a highly regulated process that involves synaptic vesicles tethering and docking to the plasma membrane followed by vesicle priming. Primed vesicles are ready to be fused in a fast Ca^2+^-dependent step (60–100µsec) triggered by membrane depolarization. This step requires a spatial and temporal precision achieved by coordinating voltage-gated calcium channels (VGCC) with the exocytotic machinery^1–3^.

Three exocytotic proteins syntaxin 1 A (Sx1A), synaptobrevin, and synaptosomal-associated-protein 25 (SNAP-25) known as the neuronal soluble N-ethylenmaleimide–sensitive factor attachment proteins receptor (SNAREs), play a key role in vesicle fusion by forming a tight four helix bundle, called the SNARE complex^4, 5^. Sx1A and SNAP-25 physically interact with the intracellular domains of VGCC, P/Q channel^6, 7^, N-type channel^8, 9^, L-type channel, Cav1.2^10^ and R-type channel^11, 12^. The effect of Sx1A and SNAP-25 on channel kinetics indicates a functional crosstalk of the two SNARE proteins with the channel, which is further modified by synaptotagmin (Syt1) (Review^13^).

The fast kinetics of the channel in a complex with Syt1 and the two SNARE proteins has been attributed to distinct physical protein–protein interactions of an exocytotic signaling unit, called the excitosome complex^10^. This heteroprotein complex is responsible for triggering vesicle fusion during membrane depolarization as shown in a reconstituted system of vesicle fusion^1, 14^.

The interface of Sx1A interaction with the channel involves two independent domains, the transmembrane domain (TMD) and the cytosolic domain^8, 10, 15–17^.

In search for the molecular basis of Sx1A interaction with the N- and L-type Ca^2+^ channels, we demonstrated that the two highly conserved cysteines, Cys271 and Cys272 in Sx1A TMD, participate in the Sx1A/Channel interaction^12, 17^. In the syntaxin 2 (Sx2) isoform the corresponding Cys271 and Cys272 are valine residues, and the cytosolic Cys145 is serine. Sx2 does not support depolarization-evoked release and fails to interact with the channel^17^. Similarly, the Sx1A double mutant Sx1A^C271V/C272V^ does not modify current amplitude, and does not support depolarization-evoked secretion in a reconstituted system^12^. The Sx1A^C271V/C272V^ mutant operates also as a dominant negative in depolarization-induced catecholamine release in chromaffin cells^18^. The interaction of Sx1A TMD with the channel led to the proposal that a signal induced by conformational change during membrane depolarization is transmitted via Sx1A TMD, triggering transmitter release within microseconds^18^.

To further explore direct interaction of the channel with Sx1A we sought to examine the close association of individual Cav1.2 and Sx1A using super-resolution imaging at the single molecule level.

Previous studies have used super-resolution microscopy to visualize Sx1A self-clustering. Sieber *et al*. have shown by a combination of far field microscopy technique the presence of Sx1A self-clustering. The clusters comprise 75 densely crowded syntaxins that dynamically exchange with freely diffusing molecules, exhibiting a diameter of 50–60 nm^19^. This resolution limit did not allow direct visualization of smaller clusters or single molecules^20^.

Using Stimulated Emission Depletion (STED) microscopy and image analysis of *Drosophila* neuro-muscular junctions, Sx1A clusters were shown to be more abundant at active zones and displayed larger sizes^21^. A higher super resolution of ∼20 nm could be achieved via direct Stochastic Optical Reconstruction Microscopy (dSTORM) that employs fluorescent probes, e.g fluorescently labeled antibodies, as immunocytochemical or chemical tags in fixed and living cells^22, 23^ showed in PC12 cells an average cluster diameter of ~93 nm^24^.

To better understand the molecular interactions that regulate neurotransmitter release, we employed two-color Photo-Activated-Localization-Microscopy (PALM) using photo-activatable mCherry (PAmCherry)^25^ and Dronpa. It allows visualizing and localization of individual molecules in intact cells with a resolution down to ~20 nm^26^. In this method photo activated (PA)-fluorescent proteins serve as genetically encoded tags that directly highlight proteins of interest, as opposed to the indirect labeling by means of antibodies in dSTORM. We used the dual-color super-resolution imaging approach^27, 28^ to determine the ultrastructural relationship between Cav1.2 and Sx1A expressed in human kidney (HEK) 293 cells. We revealed at the nano-scale level clusters of individual PAmCherry-tagged Sx1A and the Dronpa-tagged-pore forming subunit of the channel (α_1_1.2). Mutational analysis of the three-cysteine residues of Sx1A, Cys145, Cys 271, and Cys 272 revealed a correlation between nano-clustering of Sx1A/Cav1.2 and the functional interactions of Cav1.2 with the exocytotic machinery.

## Results

### PALM imaging of voltage-gated calcium channel (Cav1.2) and syntaxin 1 A (Sx1)

For imaging Cav1.2 at a molecular resolution we used Dronpa, a green photoactivatable fluorescent protein^29^ fused to the C-terminus of α_1_1.2 pore forming subunit of Cav1.2. PAmCherry^25^ was fused at the N-terminus of Sx1A and served as the second PALM color.

HEK293 cells growing in chambers (see further details in Materials and Methods) were transfected with Dronpa-tagged α_1_1.2, and the auxiliary subunit β2a or with the PAmCherry-tagged Sx1A.

**Figure 1:**
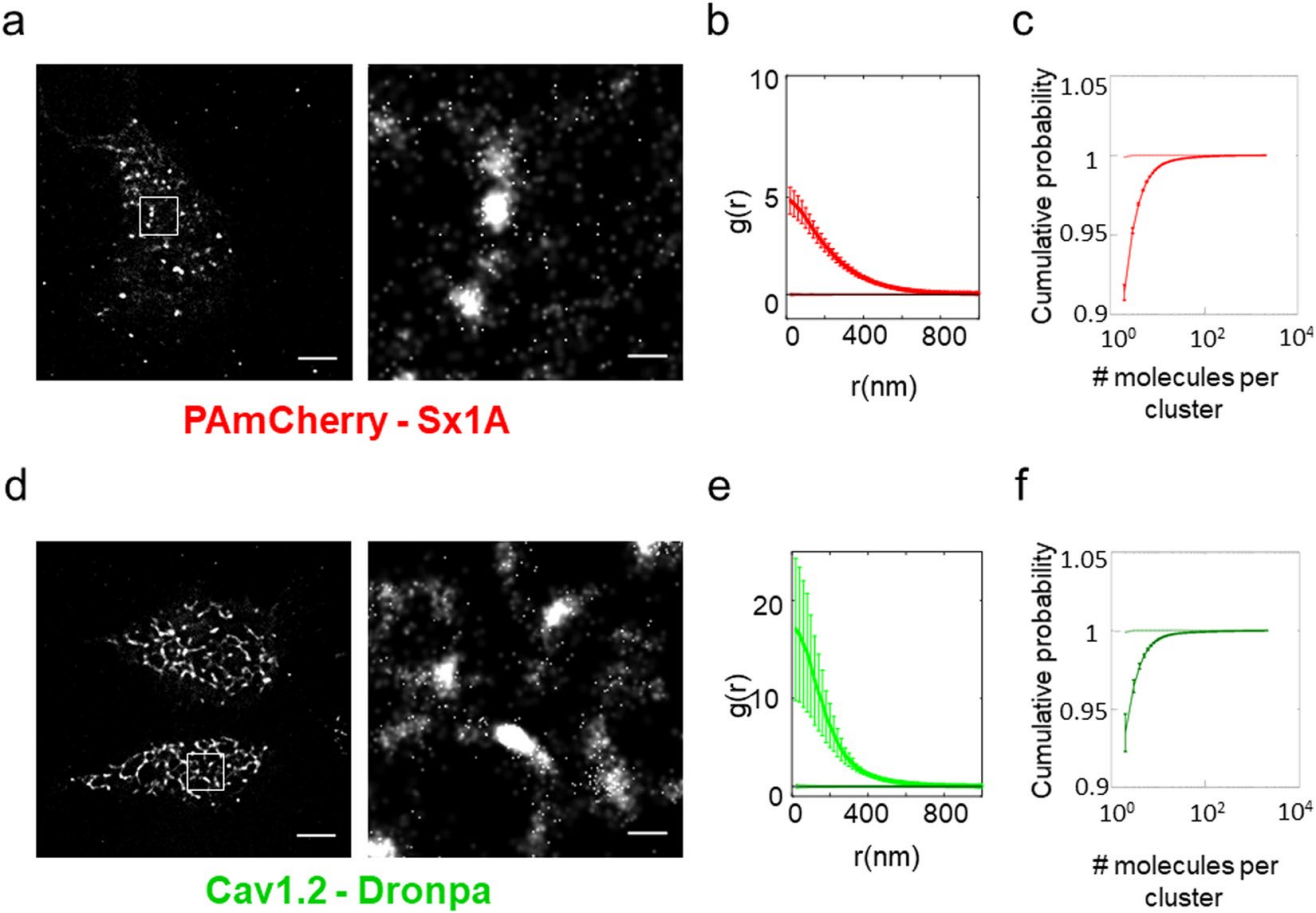
Self-clustering of Sx1 A and α_1_1.2. (**a**) A PALM image of a representative HEK293 cells expressing PAmCherry-Sx1 A. Zoom image on right shows Sx1 A clusters. Bars – 5μm (*left*) and 0.6 μm (*right*). The square brackets represent co-clusters of proteins (**b**) The average Pair-correlation function of Sx1 A in multiple cells (N = 43). Errors are SEM. (**c**) Results of a clustering algorithm to resolve individual clusters and generate cumulative size-distribution curves of the Sx1 A self-clusters. (N = 16 cells; dashed lines represent clustering of random sets; the two dashed lines overlap; see SI for further details on statistical analyses). (**d**) A PALM image of a representative HEK293 cell expressing VGCC-Dronpa (N = 43). Zoom image on right shows VGCC clusters. Bars – 5 μm (left) and 0.6 μm (right). (**e**) The average Pair correlation function of VGCC in multiple cells (N = 43). Errors are SEM. (**f**) Cumulative size-distribution curves of the VGCC self-clusters (N = 16 cells).

Forty-eight hours after transfection cells expressing the proteins were imaged by PALM (Fig. 1). We observed self-clusters of Cav1.2 and Sx1A organized at the plasma membrane (PM) (Fig. 1a,d). The self-clustering was quantified and compared to a random Poisson process using the second order statistics of univariate pair correlation function (PCF)^30^.

The Sx1A PCF appeared ~4 times lower than the Cav1.2 PCF, while both were significantly higher than the PCF model of a Poisson process (Fig. 1b,e). To further quantify the number of molecules per cluster of either Sx1A or Cav1.2, we employed a published clustering algorithm^31^ with a distance threshold of 30 nm (see algorithm details and parameters in Supplementary information).

The cumulative distribution of number of molecules per cluster of Cav1.2 or Sx1A is shown in Fig. 1c,f (bold lines with error-bars). Very small clusters of dimers (~92–93%) and trimers (~3–4%) appeared to dominate the size distribution of clusters for either protein. The larger clusters may contain up to several dozens of molecules, but constitute only a small fraction of the identified clusters. As with the PCF analyses, these distributions were compared and found significantly different from distributions due to the Poisson process (dotted lines). Thus, we conclude that both Cav1.2 and Sx1A are organized in nano-clusters of dimers and trimers, while larger clusters are of lower frequency. Both Sx1A and Cav1.2 showed a unimodal distribution of the clusters’ area (Fig. S2a,d), which was skewed and contained a small fraction of large clusters. Note that in this latter analysis of the clusters’ area, monomers and dimers were not considered as their area cannot be well defined.

We next used the clustering analyses to study the effect of protein expression level on their extent of self-clustering. The protein expression level was monitored by the total abundance of detected molecules at the PM of the cells, as counted by our PALM analyses. Figure S2b,e show the average cluster size of Sx1A and Cav1.2 in copy number with Pearson coefficients of 0.17 and 0.19, respectively. Figure S2c,f show the average cluster area of these proteins with Pearson coefficients of 0.08 and 0.02, respectively. Thus, we observed no significant dependence of the average cluster size on the expression level.

### Cav1.2 channels co clusters with Sx1A at the plasma membrane

Sx1A and the α_1_1.2 subunit of Cav1.2 interact with each other both through the cytosolic and the trans membrane domains^10^. To examine nanometer-scale correlations between the two membrane proteins at a molecular resolution we used the two-color PALM. The utility of PAmCherry as an intracellular probe was previously established for detecting PALM images of transferin receptor clusters at 200 nm resolution or clathrin light chain at 25 nm resolution^25^. The reversibly photo- activatable fluorescent protein Dronpa was used to study dynamics of SNAP-25 cluster domains^32^.

**Figure 2:**
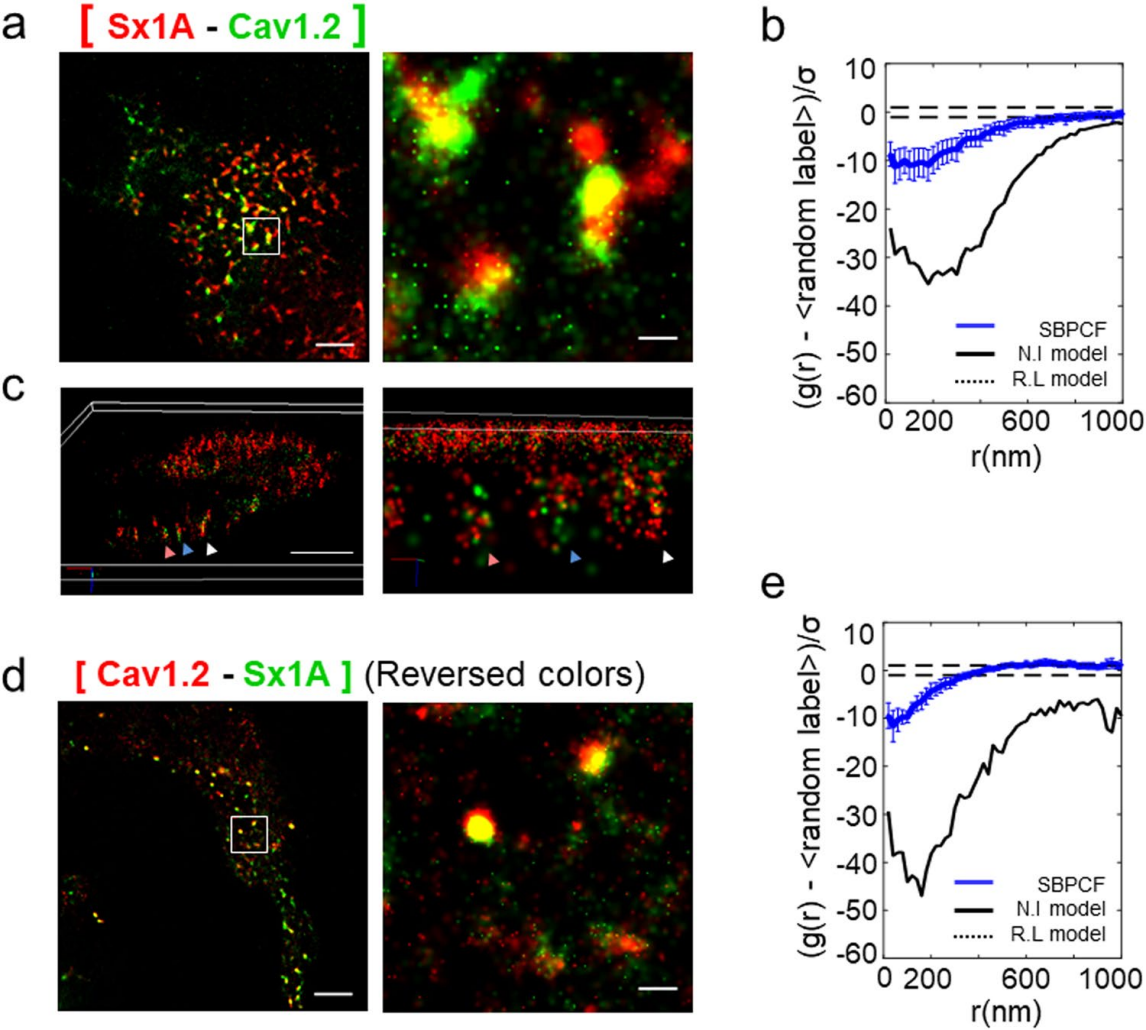
VGCC and Sx1 A form a complex. (**a**) Two-color PALM imaging of HEK293 cells expressing VGCC-Dronpa and Sx1A-PAmCherry. Bars – 5 μm (*left*) and 0.6 μm (*right*). The square brackets represent co-clusters of proteins (**b**) Standardized bivariate PCF of VGCC-Dronpa and Sx1A-PAmCherry (*blue*) (N = 43), compared to the 95% confidence interval of a Random labelling model (black dotted lines at 1 and −1) and a model of no interaction (NI; black solid line) (**c**) Three-dimensional PALM imaging of HEK293 cells expressing VGCC-Dronpa and Sx1A-PAmCherry. Bar – 16 μm. The PM of a representative cell is shown (N = 10). Individual clusters are highlighted by colored arrowheads (**d**) Two-color PALM imaging of HEK293 cells expressing Dronpa-Sx1 A and VGCC-PAmCherry. Bars – 5 μm (*left*) and o.6 μm (*right*) (**e**) Standardized bivariate PCF of Dronpa-Sx1 A and VGCC-PAmCherry (*blue*) (N = 18), compared to the 95% confidence interval of a Random labelling model (black dotted lines at 1 and −1) and a model of no interaction (NI; black solid line).

HEK293 cells were co-transfected with the Dronpa-tagged α_1_1.2 subunit, the unlabeled auxiliary subunit β2a and PAmCherry-tagged Sx1A. Forty-eight hours after transfection we observed two-colored (<100 nm) α_1_1.2 and Sx1A clusters (yellow) co-localized at the plasma membrane (Fig. 2a; Fig. S5a).

For analyzing protein co-clustering, we employed a modified bivariate second-order analysis, termed ‘standardized bivariate PCF’ (SBPCF)’ described here for the first time (see details in Supplementary information and Fig. S1a,b). This analysis enables comparing the bivariate PCF for multiple cells, in contrast to previous studies where bivariate PCFs were shown only for single representative cells^33, 34^. The SBPCF analysis demonstrates that Cav1.2 and Sx1A co-cluster and integrate into mutual complexes at the PM of the cells **(**Fig. 2b). Previously, we have introduced an alternative measure for comparing bivariate molecular interactions that we termed the ‘Extent of Mixing’ (EOM)^33, 34^ (see details in Supplementary information and Fig. S1a,c). This measure typically yields PCF values that range between 0 for non-interacting species, and 1 for closely interacting species. It provides an intuitive indication of the extent of interaction between two species. Note that this measure is normalized, and thus, absolute correlation values cannot be compared. For better interpretation of our results, we provide this complementary measure along the SBPCF results for each molecular interaction under study (see Fig. S3a for the Cav1.2-Sx1A interaction).

To explore the Sx1A-PAmCherry and Cav1.2-Dronpa organization within their mutual clusters, we imaged the cells using 3D PALM (see details in Materials and Methods). We observed that Sx1A and Cav1.2 were mixed homogenously within the clusters, showing no distinct organization pattern (Fig. 2c).

Next, we reversed the tags of the proteins and used PAmCherry-tagged Cav1.2- and Dronpa-tagged Sx1A-and compared the PALM images. As shown, the co-clustering patterns of the reversed colors and SBPCF-statistics were similar, confirming lack of effect of the fluorescent protein tags on protein clustering (Fig. 2d,e).

### Cav1.2 does not cluster with proteins unrelated to exocytosis

**Figure 3:**
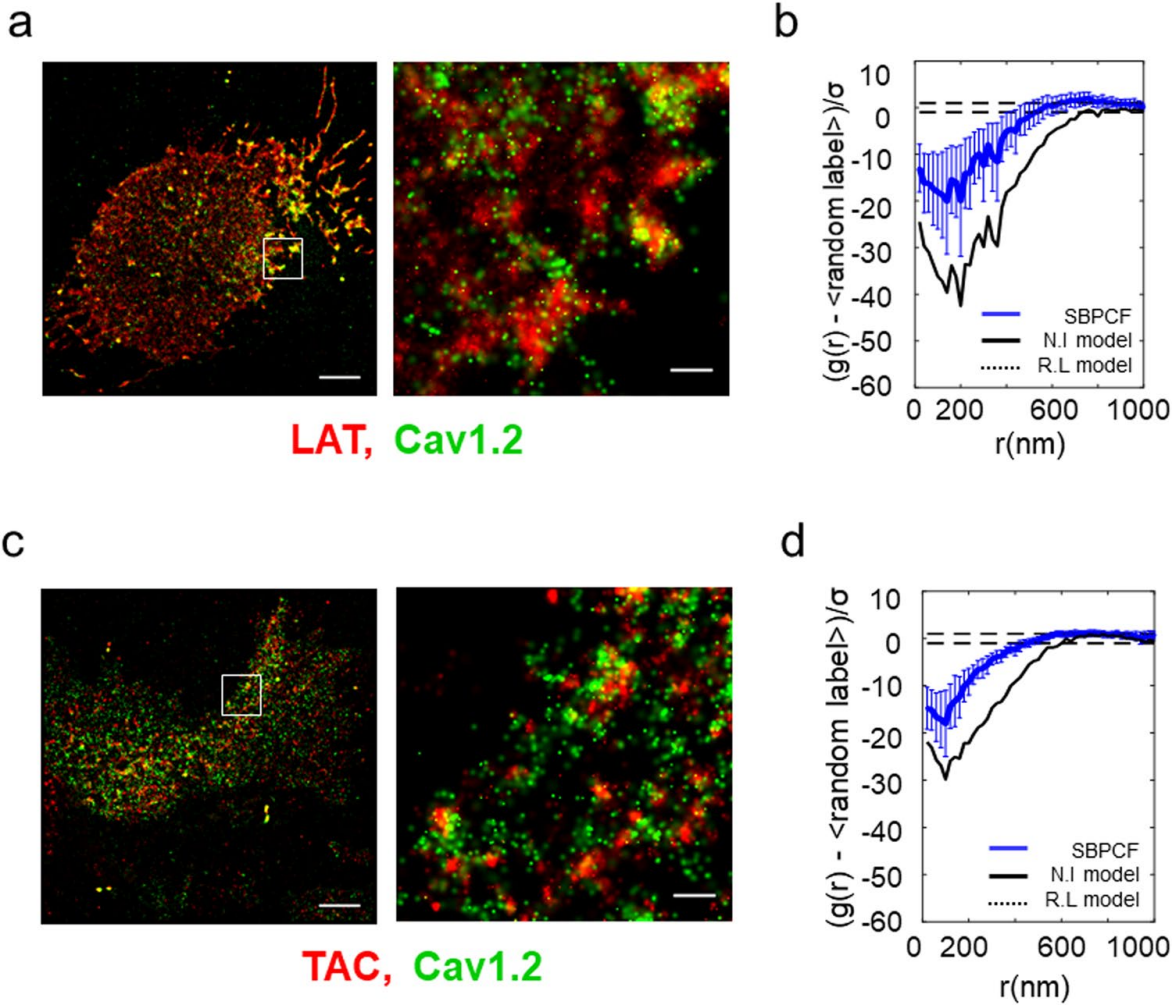
Cav1.2 does not form co-clusters with LAT or TAC. Two-color PALM imaging of HEK293 cells expressing Cav1.2-Dronpa either (**a-b**) LAT-PAmCherry or (**c-d**) TAC-PAmCherry. Bars – 5 mm (*left*) and 0.6 mm (*right*). Standardized bivariate PCF of Cav1.2-Dronpa and LAT-PAmCherry (N = 17) (**b**) or TAC-PAmCherry (N = 15) (**d**) compared to the 95% confidence interval of a Random labelling model (black dotted lines at 1 and −1) and a model of no interaction (NI; black solid line).

To further validate the observed PALM interaction between Cav1.2 and Sx1A, we conducted negative controls using similar imaging of Cav1.2 with proteins lacking contact with the channel and which are unrelated to the exocytotic machinery (Fig. 3). For that, α_1_1.2-Dronpa was co-transfected with either PAmCherry-tagged-Linker for activation of T-cells (LAT) (Fig. 3a,b), or PAmCherry-tagged TAC (TAC is CD25, the human IL-2 receptor alpha subunit^31^) (Fig. 3c,d). These two proteins did not co-cluster with Cav1.2. Their SBPCF curves indicated interactions that were much closer to the black solid line at the bottom, in comparison to the interaction indicated between Cav1.2 and Sx1A (Fig. 3b,d). According to the model, the solid black lines of the SBPCF indicate no interaction. We attribute the apparent residual interaction of Cav1.2 with either LAT or TAC to patterning of the PM (See also Fig. S3b,c). PM patterns often show voids, i.e. black regions at the cells’ footprint devoid of molecules.

### Sx2 displays no clustering with the channel

**Figure 4:**
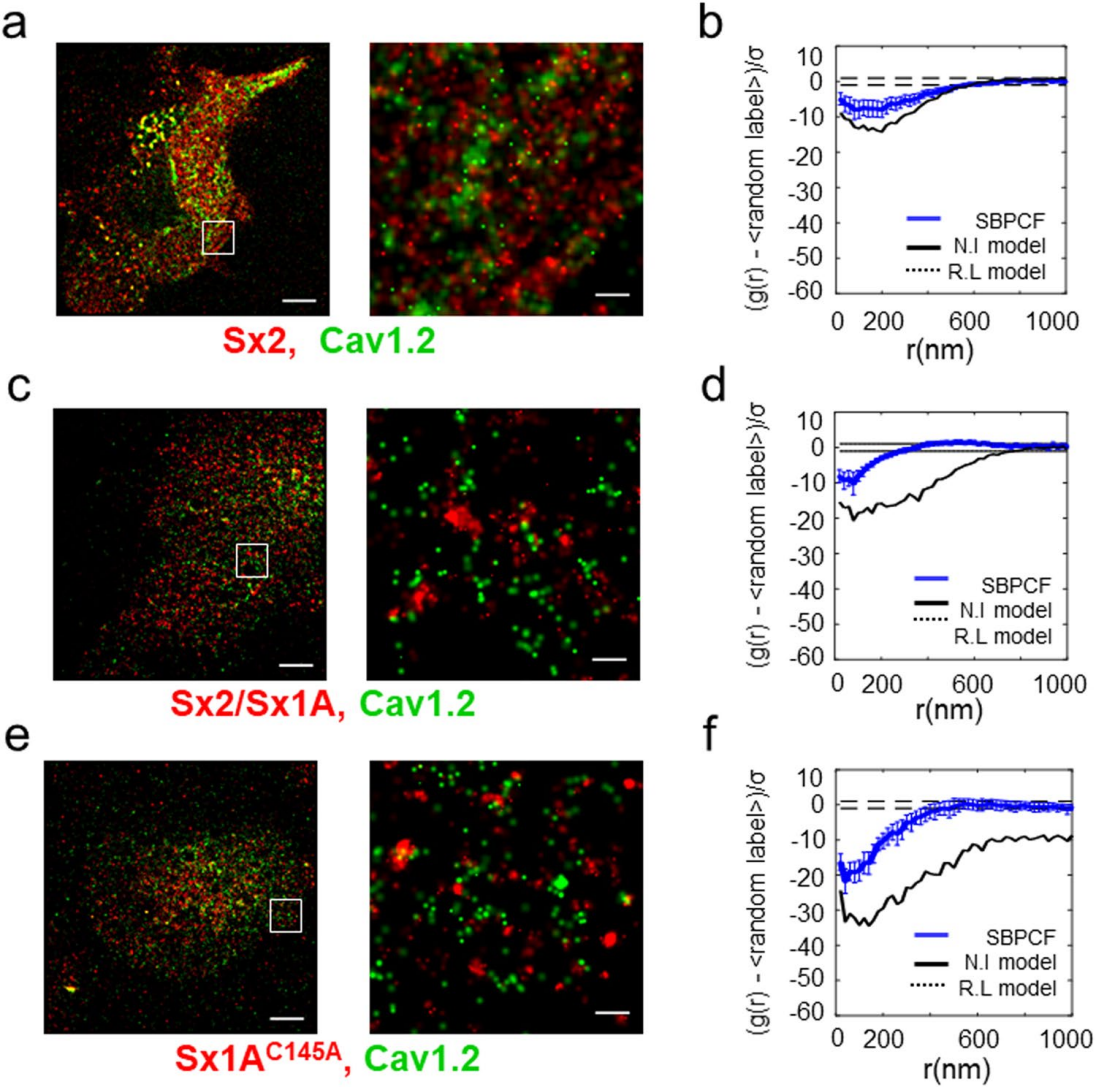
Cys145 in Sx1 A cytosolic domain is essential for co-clustering with Cav1.2. (**a**) Two-color PALM imaging of HEK293 cells expressing Cav1.2-Dronpa with PAmCherry-Sx2. Bars – 5 µm (*left*) and 0.6µm (*right*). SBPCF represented in (**b**) (N = 26) (**c**) Two-color PALM imaging of HEK293 cells expressing Cav1.2-Dronpa with PAmCherry-Sx2/Sx1 A. Bars – 5 µm (*left*) and 0.6 µm (*right*). SBPCF represented in (**d**) (N = 17) (**e**) Two-color PALM imaging of HEK293 cells expressing Cav1.2-Dronpa with PAmCherry-Sx1 A^C145A^. Bars – 5 µm (*left*) and 0.6µm (*right*). SBPCF represented in (**f**) (N = 34).

Sx2 is an isoform of 79% sequence homology to Sx1A that does not support exocytosis, and does not interact with the channel, as previously shown in the *Xenopus* oocytes heterologous expression system^12, 17^. To test whether nano-clustering of Sx1A with α_1_1.2 correlates with functional interaction we examined cluster formation of Sx2 with α_1_1.2. The PAmCherry-tagged Sx2 was co-transfected with α_1_1.2-Dronpa and PALM imaging was detected in HEK293. As opposed to cells expressing wt PAmCherry-tagged Sx1A, no clusters with Dronpa-tagged α_1_1.2 with Sx2 were detected (Fig. 4a,b; Figs S3d, S5b). These results are consistent with Sx2 inability to support exocytosis, shown by capacitance measurements of reconstitution secretion^12^, or modify Cav1.2 kinetics, as opposed to the negative impact of Sx1A^17^.

Hence, Sx2 failure to co-cluster with the channel strongly supports a correlation between functional interactions and nano-clustering formation.

The transmembrane domain and the cytosolic domain of Sx1A differently modify channel kinetics^10, 12, 17^. To examine the exclusive contribution of the cytosolic interaction on co-clustering with the channel, we used Sx2/Sx1A chimera, constructed by exchanging the Sx1A and Sx2 cytosolic-domains^17^. The Sx2/Sx1A chimera did not form clusters with the channel as shown in Fig., 4c,d; Fig. S5c, confirming a major role of the Sx1A cytosolic domain in the assembly with the channel (See also Fig. S3e). The absence of channel co-clustering with Sx2 or with the Sx2/Sx1A chimera, is correlated with the lack of functional interactions of these two proteins with the channel, as previously demonstrated^17^.

### The cytosolic cysteine of Sx1A is essential for co-clustering with the channel

Sx1A has a single cysteine residue in its cytoplasmic domain, Cys145, which is conserved in neuronal Sx1A isoforms, and absent from most non-neuronal isoforms^35^. In view of the importance of the Sx1A cytosolic domain to nano-cluster formation we further examined the selective and specific role of the cytosolic Cys145 to the crosstalk of Sx1A and the channel.

No clustering of Sx1A^C145A^ mutant with Cav1.2 was detected and the SBPCF curve was similar to Cav1.2 with the non-related molecules LAT and TAC (Fig. 4e,f see also related curves in Fig. S3b–f). These results highlight the critical impact of the single cytosolic Cys145 of Sx1A in clustering with the channel (Fig. S5d).

### Mutated Sx1A-TMD Cys271 and Cys272 modify Sx1A clustering with the channel

The Sx1A double mutant Sx1A^C271V/C272V^ (CC/VV) abolishes the negative impact of Sx1A on Cav1.2, Cav2.1, Cav2.2, and Cav2.3 current amplitude^12, 16, 17, 36^. This highly conserved Cys271 and Cys272, which correspond to Val271 and Val272 in Syx2, appeared to be critical in transmitting a signal from the channel to the exocytotic machinery^7, 12, 16, 17^.

**Figure 5:**
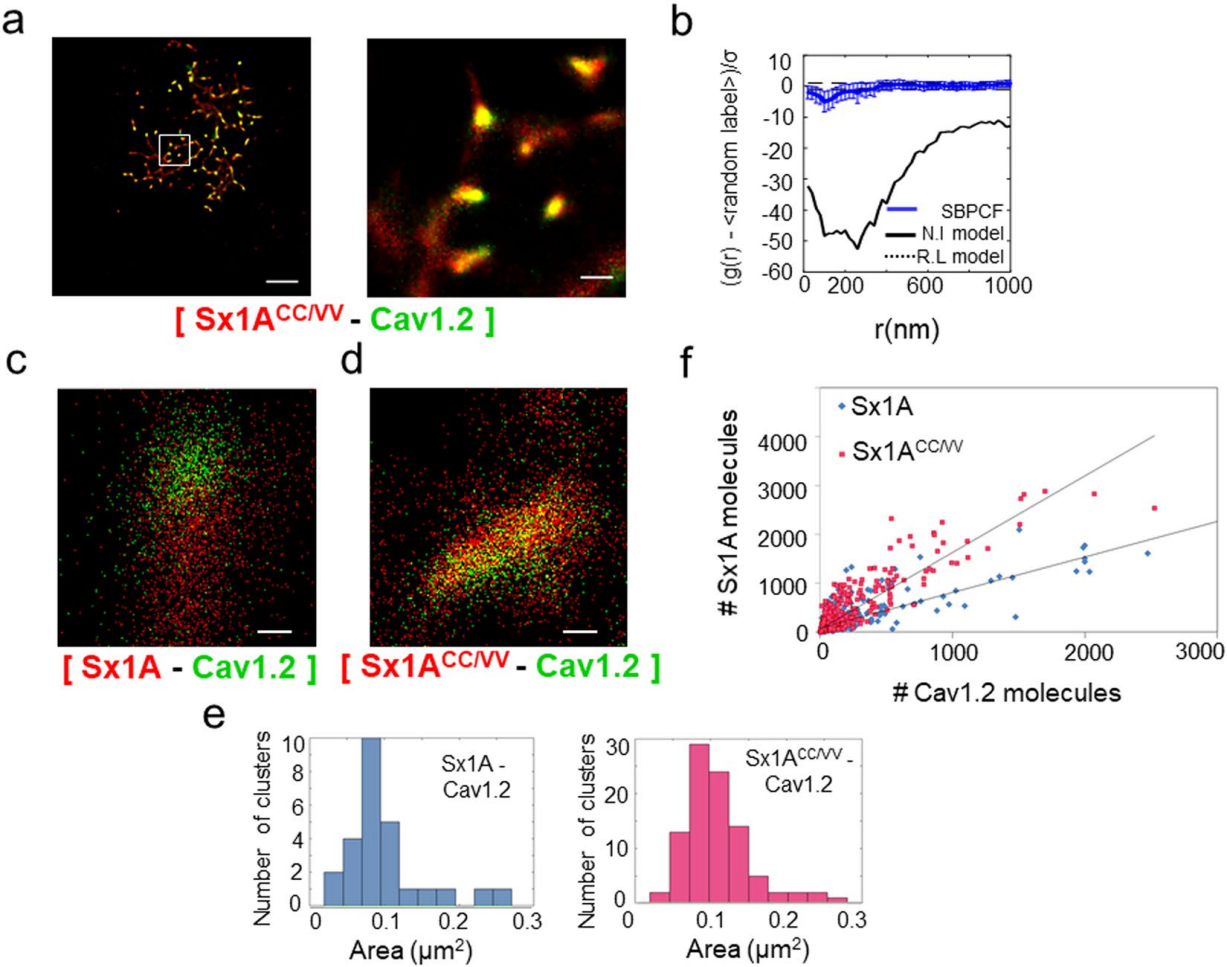
An increased ratio of non-functional Sx1 A^CC/VV^ mutant to Cav1.2 in hetero-nano-clusters. (**a**) Two-color PALM imaging of HEK293 cells expressing Cav1.2-Dronpa with double-mutant PAmCherry-Sx1 A^C271V/C272V^. Bars – 5 µm (*left*) and 0.6µm (*right*). SBPCF represented in (**b**) (N = 22). Single cluster analysis was performed on cells represented in Fig. 2a (wt interaction) and Fig. 5a (double-mutant Sx1 A^C271V/C272V^). Zoom on chosen cluster in cells expressing wt or mutant Sx1A (**c**,**d** respectably). Bars – 0.2 µm (**e**) The statistics of clusters area in one cell is displayed by graphs. Clusters of wt Sx1 A and Cav1.2 display a mean area of 0.075 µm^2^ (*left panel* and the double-mutant Sx1 A^C271V/C272V^ and Cav1.2 display a mean area of 0.1 µm^2^ (*right panel*) (**f**) Proportion of red (PAmCherry-Sx1 A) to green (Cav1.2-Dronpa) in clusters (blue for a wt Sx1 A and red for a mutant Sx1 A^CC/VV^). The proportion of red to green molecules in wt is ~0.7 ± 0.02 (y = 0.7315x + 76.508, R² = 0.6738) and ~1.6 ± 0.03 for a double-mutant Sx1 A^C271V/C272V^ (y = 1.5681x + 58.944, R² = 0.7776).

The impact of Cys271 and Cys272 on clustering with the channel was explored by co-expressing the Sx1A double-mutant PAmCherry-tagged Sx1A^C271V/C272V^ (CC/VV) with Dronpa-tagged α_1_1.2 in HEK293 cells. Clusters of Sx1A^CC/VV^ mutant with the channel were found (Fig. 5a **)**.

The resultant SBPCF curves resided close to the 95% confidence interval due to the model of random labeling, indicating that the proteins mixed more homogeneously, and were closely associated in mutual clusters (Fig. 5b; see Supplementary Information; Fig. S3g).

As opposed to PALM images of individual clusters of Cav1.2-Dronpa and wt -PAmCherry-Sx1A (Fig. 5c), the Cav1.2-Dronpa and PAmCherry-Sx1A^CC/VV^ (Fig. 5d) showed a tighter interaction and appeared more homogeneously associated. In Fig. 5f, the molecular ratio of Sx1A (red) *vs* Cav1.2 (green) in clusters was comprised of ~0.7:1 red to green, while for Sx1A^CC/VV^ mutant the proportion was changed to ~1.6:1 (Fig. S6a,b; see analysis details in the Supplementary information). Still, the mean area of the co-clusters remained almost the same (peaking at around 0.08 µm^2^) (Fig. 5e).

### Correlation of Cys oxidation/reduction with Sx1A/α_1_1.2 channel clustering

**Figure 6:**
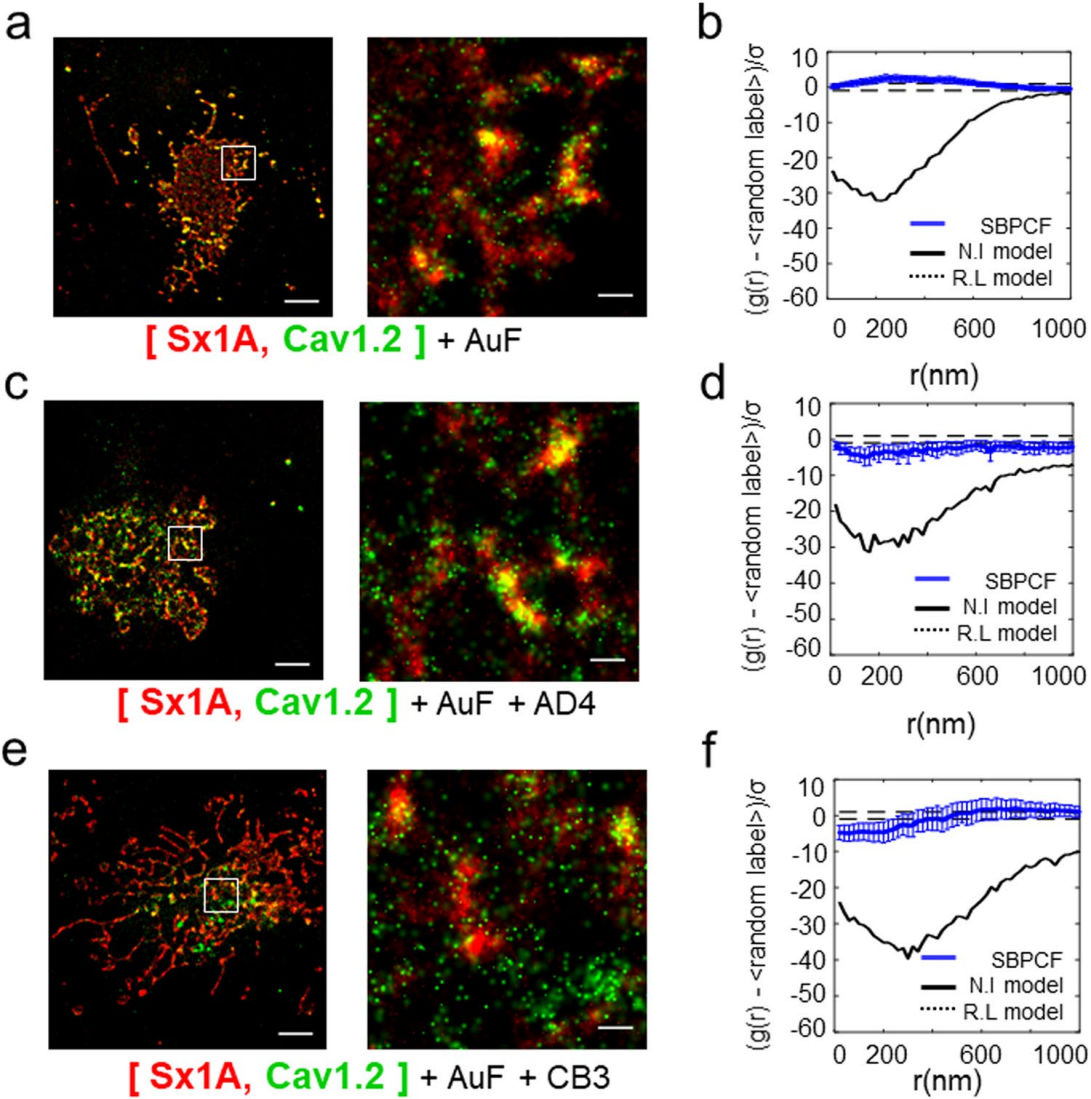
Oxidation affects the Sx1 A/channel ratio in hetero-clusters. (**a**) Two-color PALM imaging of HEK293 cells expressing VGCC-Dronpa with Sx1A-PAmCherry followed by auranofin (10 µM) treatment. Bars – 5 µm (*left*) and 0.6 µm (*right*). SBPCF represented in (**b**) (N = 35) (**c**) Two-color PALM imaging of live HEK293 cells expressing α_1_1.2-Dronpa with Sx1A-PAmCherry followed by auranofin (10 µM) and AD4 (1mM) treatments. Bars – 5 µm (*left*) and 0.6 µm (*right*). SBPCF represented in (**d**) (N = 34) (**e**) Two-color PALM imaging of live HEK293 cells expressing Cav1.2-Dronpa with Sx1A-PAmCherry followed by auranofin (10 µM) and CB3 (150 mM) treatments. Bars – 5 µm (*left*) and 0.6 µm (*right*). SBPCF represented in (**f**) (N = 34).

Modulation of catecholamine release and channel activity by Sx1A  is impaired under oxidation conditions and is attributed to the highly conserved, redox sensitive Cys residues within Sx1A TMD^37, 38^. Here, we examined the effect of oxidation on Sx1A/channel co-clustering. We imaged cells after incubation with 10µM auranofin, a thiol-oxidizing reagent for 30 min. Analysis of the two-color-PALM imaging revealed a higher extent of association of Sx1A with the channel, similar to the Sx1A^C271V/C272V^ mutant (Fig. 6a). Also the SBPCF statistics under these oxidized conditions was similar to that observed with the Sx1A TMD-mutant (Fig. 6b **;** Fig. S3g,h).

The oxidation by auranofin can be reversed by exposure to reducing conditions e.g thiol-reagents such as N-acetylcysteine amide (NAC-amide; AD4)^39^ or thioredoxin-mimetic peptides TXM-CB3^18, 37^. Following exposure to AuF, the cells were washed, and then pre-incubated for additional 30 min with 1mM AD4 (Fig. 6c **;** Fig. S3i) or 100 µM TXM-CB3 (Fig. 6e **;** Fig. S3j). Two color PALM images taken after washing out the reducing reagents revealed a partial yet significant reversal of the Sx1A/Cav1.2 co-clustering observed in the presence of AuF (Fig. 6d,f **;** Figs S3i,j, S6c **)**.

The effect of oxidation/reduction conditions on the Sx1A/channel co-clustering, which was similar to the effect for the Sx1A TMD mutation, further supports Cys271 and Cys272 involvement in cluster formation and interaction with the channel.

### Self-Clustering of Sx2, and Sx1A^CC/VV^, Sx1A^C145A^

To explore self-clustering of Sx1A in comparison to Sx2, and Sx1A^C271V/C272V^, we expressed the corresponding PAmCherry-tagged Sx1A mutants. The one-color PALM imaging showed no significant difference in the size of clusters formed by Sx1A, Sx2 or Sx1A^CC/VV^ (comparing Fig. S4a–f and Fig. 1a–c). Interestingly, the extent of Sx1A^C145A^ mutant self-clustering appeared higher (g_12_(0) ~7) than that of Sx1A wt (g_12_(0) ~5) (comparing Fig. 1a–c and Fig. S4jJ–l **)**. Clustering of Cav1.2 and Sx1A seems to be correlated with functional and physical interaction between these proteins^10, 15, 17^. It appears that the sequence at the TMD does not affect self-clustering, as opposed to alterations observed in channel co-clustering with Sx1A and Sx1A^CC/VV^. Similarly, the extent of self-clustering of Sx2/Sx1A chimera appeared higher compared to the self-clustering of Sx1A, Sx2 and Sx1A^CC/VV^ each alone (comparing Fig. 1a–c and Fig. S4a–f to Fig. S4g–l). This effect of the cytosolic domain of Sx1A seems limited, as it did not significantly modify the overall clustering distributions, in terms of either the clusters’ physical size (Fig. S4b,e,h,k) or number of molecules per cluster (Fig. S4c,f,j,i). Variations in expression levels among the different mutants might affect the self-clustering of these molecules, while having a limited (or no) effect on the co-clustering analyses^34^. In the present study we imaged transiently transfected cells, which minimizes bias due to expression level as determined by overall intensity, and would limit the interpretation of differences in self-clustering.

## Discussion

In this study we used the dual-color super resolution-imaging approach (PALM) to monitor in single molecule detail L-type voltage-gated calcium channels (Cav1.2) nano-clustering with Sx1A, Sx2 isoform, and Sx1A mutants.

### Sx1A forms ~1:1 cluster with Cav1.2

Unlike Sx1A self-clustering in the cell membrane, which was extensively examined in several high-resolution imaging of techniques^19–21, 24, 28, 40, 41^, co-clustering of Sx1A with Cav1.2 was not examined. Our major goal was to explore at the single molecule level the Sx1A interaction with the pore forming subunit of Cav1.2 using PALM super resolution microscopy.

Co-localization of Sx1A and Cav1.2 was examined in HEK293 transfected with PAmCherry-tagged Sx1A (red) and Dronpa-tagged α_1_1.2 (green). The two-color PALM super resolution imaging showed that Sx1A and Cav1.2 generated co-clusters with nano-scale sizes, below the diffraction limit of light. Cluster analysis revealed ~1:1 ratio Sx1A/α_1_1.2 clusters (yellow), which was not affected upon switching the fluorophores to PAmCherry-tagged α_1_1.2 and Dronpa-tagged Sx1A. These hetero-protein clusters indicate Cav1.2 interaction with Sx1A at the single molecule level, lending further credence for a functional crosstalk by these two proteins (review^1^).

### Sx1A clustering with the channel requires Sx1A cytosolic Cys145 and does not form clusters with Sx2 or Sx2/Sx1A chimera

Sx1A has a single cysteine residue in the cytoplasmic domain, Cys145, which is highly conserved in the neuronal Sx1A isoforms, and is absent from non-neuronal isoforms^42^.

The PAmCherry-tagged Sx1A^C145A^ mutant unlike wt Sx1A revealed no clustering with α_1_1.2, suggesting that the mutation might have entailed a change in intermolecular interactions, preventing cluster formation.

This dramatic effect on co-clustering indicates the importance of Sx1A-cytosolic domain in general and Cys145 in particular. Previously, recombinant-protein binding assays have shown that the Sx1A cytosolic-domain binds to the cytosolic II-III loop-domain of α_1_2.2^6^ and α_1_1.2^10^. Furthermore, the recombinant II-III loop inhibits insulin secretion in pancreatic islets, most likely through competition with wt channel on interaction with Sx1A cytosolic-domain^10, 43^.

The importance of Cys145 to co-clustering with the channel was further demonstrated using Sx2 isoform in which Cys145 is a Ser residue. We also used a Sx2/Sx1A chimera, constructed by exchanging the cytosolic domain of Sx1A with the Sx2 cytosolic-domain. Both PAmCherry-tagged Sx2 and the PAmCherry-tagged Sx2/Sx1A chimera, revealed no co-clustering with Dronpa-tagged α_1_1.2. This result further confirms the role of Cys145 in channel modulation and highlights the crosstalk between Sx1A and Cav1.2 at the single molecule level. The absence of co-clusters with Sx2 or Sx2/Sx1A chimera appears to coincide with their inability to modulate current amplitude^12, 17^. More recently, Sx2 was shown to act as an inhibitory SNARE protein for insulin granule exocytosis^44^.

### The effect of Sx1A TMD in Sx1A clustering with Cav1.2 is determined by channel mutants and by perturbing oxidation-reduction

The functional coupling between the channel and Sx1A is essential for triggering transmitter release, and requires the presence of the two highly conserved TMD Sx1ACys271 and Cys272^18^. The two-color PALM imaging analysis revealed ~2:1 ratio nano-clusters of Sx1A^CC/VV^/α_1_1.2, compared to the ~1:1 ratio observed in nano clusters of wt Sx1A/α_1_1.2. The increase in the number of Sx1A-mutant molecules associated with the channel coincides with the Sx1A^CC/VV^ inability to modify Cav1.2 kinetics. Such an overcrowding effect could perhaps be responsibe for the non-functional complex that fails to modify current amplitude and to support exocytosis^12, 17, 36^. Sx1A^CC/VV^ acts also as dominant-negative in depolarization-induced catecholamine release in chromaffin cells^18^. Alternatively, an over-crowding of Sx1A could impair recruitment by SNAP-25, a necessary step for regulating Ca^2+^ channels^15, 45^. We conjecture that Sx1A/channel clusters (~1:1) might represent an active molecular complex since the increased number of Sx1A molecules in the nano-cluster is correlated with a loss of synaptic activity.

Oxidizing reagents alter Sx1A interaction with the channel and disrupt exocytosis (Review^46^). Changes in channel kinetics were demonstrated in *Xenopus* oocytes treated with the vicinal selective thiol-oxidizing reagent phenyl-arsene-oxide (PAO)^36^ or with AuF^37^. Loss of Sx1A interaction with the channel under oxidizing conditions virtually abolishes catecholamine release in chromaffin cells and insulin release in insulinoma cells. Loss of activity was  reversed following the addition of thiol reducing reagents such as N-acetylcysteine, N-acetylcysteine amide (AD4)^39^, or the thioredoxin mimetic peptides, TXM-CB3, TXM-CB4 or TXM-CB6^18, 37, 38^.

Our PALM results show that oxidation by AuF increased Sx1A co-clustering with the channel, while subsequent exposure to the thiol-reducing reagents like AD4 or TXM-CB3, partially reversed the co-clustering ratio.

Compromised channel interaction with Sx1A and impaired exocytosis caused under oxidative stress conditions, were observed also by mutating the  Sx1A TMD Cys 271 and 272, and were attributed to a change in the redox state of the -SH groups of the TMD cysteine residues. Our PALM imaging analysis that shows an increase in Sx1A/channel co-clustering under oxidizing conditions, as well as by the Sx1A^C271V/C272V^ double mutation, concurs with crippling of the physiological activity.

A partial reversal of AuF induced clustering by AD4 or TXM-CB3 might have an alternative explanation. Nevertheless, since AuF has been shown to react with Sx1A Cys271 and Cys272^18^, the reducing activity of both AD4 and TXM-CB3 would appear to be the most likely mechanism involved in the reversal of the AuF-oxidizing effects.

*In summary*, biochemical and physiological studies have previously shown an interaction between voltage-gated calcium channels and Sx1A. Here we demonstrate, at the single molecule level, that PAmCherry-tagged-Sx1A co-clusters with Dronpa-tagged Cav1.2 and is dependent on the cytosolic Sx1A Cys145. The functional coupling of Sx1A with the channel and depolarization-triggered exocytosis are highly sensitive to the redox state of the Sx1A TMD Cys271 and Cys272. We found an increase in the ratio of Sx1A molecules vs. Cav1.2 molecules in their co-clusters by mutating Cys271 and Cys271. We also found an increase in the extent of co-clustering under oxidizing conditions. Thus, the higher ratio and the higher extent of co-clustering appears to correlate with a decrease in functional activity. Although our PALM data, similar to other PALM studies, do not reflect binding of proteins, the clustering of the channel with Sx1A at the single cell level, observed by the PALM imaging, supports previous biochemical and electrophysiological studies. In these studies Sx1A has been shown to interact both physically and functionally with channel^1, 10, 15^. The involvement of Cys271 and Cys272 was also shown in biochemical and functional assays^17, 36^. Our models are indicative of a direct binding of Sx1A to the channel and are based on both PALM data and biochemical studies (Figs S5 and S6).

Our results pave the way for exploring  the impact of other relevant exocytotic proteins on co-clustering with the channel and for correlating super-imaging results with channel kinetics and evoked-transmitter release.

## Materials and Methods

### Cell culture and treatment

Human Embryonic 293 kidney (HEK293) cells were cultured at 37 °C, 5% CO_2_ in Dulbecco’s modified Eagle’s medium (DMEM) supplemented with 10% fetal bovine serum, penicillin, and streptomycin. Undifferentiated HEK293 cells were plated at a density of 7.5 × 10^4^/cm^2^ on poly lysine-coated µ-chambers and incubated for 24 h.

### Transfection

The cells were transfected using Lipofectamin 3000 transfection reagent (Life Technologies) according to the manufacturer’s instructions. Transiently transfected cells were monitored for positive expression of PAmCherry or Dronpa proteins and imaged within 48 hr from transfection.

Cells were fixed by 2.4% Paraformaldehyde (PFA) prior imaging.

### Drug treatment

Auranofin (triethylphosphine (2,3,4,6-tetra-*O*-acetyl-β-1-d-thiopyranosato-*S*) gold(I)) was from Enzo Life Sciences, Shoham, Israel;.

Auronofin (10 µM) was added prior to imaging (48hr post transfection) for 30 min at 37 °C^37^. 1mM AD4 (NACA; NAC-amide; Novetide) or 150 mM TXM-CB3 was added after Auranofin treatment to reverse conditions.

### Constructs

The preparation of the mutants of Sx1A: Sx1A^C145A^, Sx1A^C271V/C272V^, and the Sx2/Sx1A chimera by exchanging the transmembrane region with the cytoplasmic domains of Sx1A and Sx2 have been previously described^17, 18^.

The photoactivable (PA) fluorescent fusion proteins of each of the syntaxin constructs was performed by PCR using the following primers, which added a BsrG1 and a BglII site at the ends^31^.

### Sx1A primers

Forward AAAATGTACAAGATGAAGGACCGAACCCAGGAGCTCGCG

Reverse AAAAAGATCTCTATCCAAAGATGCCCCCGATGG

### Sx 2 Primers (RAT NM_012748.1)

Forward AAAATGTACAAGATGCGGGACCGGCTGCCGGAC

Reverse AAAAGATCTTCATTTGCCAACCGTCAAGCC

The PCR products were cut with BsrG1 plus Bgl II and ligated to the same sites in the vector, PAmCherry to create a fusion protein with the cherry moiety at the N -terminus.

### α_1_1.2 primers

The normal human α_1_1.2 channel subunit was a generous gift of Dr. R. Splawsky. All mutagenesis were performed using the Quick Change procedure^47, 48^.

*Dronpa α*_1_1.*2* The human clones were copied by PCR adding an Nhe I site at each end, plus Bam HI sites at the end using the following primers:

Forward: AAAGCTAGCATGGTCAATGAGAATACGAGGATG

Reverse: AAAGCTAGCAGGCTGCTGACGTAGACCCTGCT

The PCR product was cut and transferred to the same site in a vector, LAT-Dronpa, to create a fusion protein with the Dronpa moiety at the C terminus.

*PAmCherry α*_*1*_ *1*.*2* The Human α_1_1.2 was sub-cloned into the cherry vector pM-C1- Cherry using the following primers to perform PCR of the normal channel.

Forward primer -AAAAAACTCGAGATGGTCAATGAGAATACGAGGAT

Reverse primer -AAAAAAGGATCCGGCAGGCTGCTGACGTAGACCCTGC

An Xho I site was thereby created at the 5′ end and a BamHI site at the 3′ end. The cut PCR fragment was introduced into the corresponding sites of the C1 vector to create a channel-PAmCherry fusion protein. Both α_1_1.2 clones harbored the nifedipine resistance mutation T1036Y in ref. 49. The LAT-PAmCherry and TAC-PAmCherry plasmids were available for this study from a previous study^33^.

### PALM microscopy

Two-color PALM imaging was performed on a total internal reflection (TIRF) microscope (Nikon). Imaging in TIRF mode served to visualizes molecules at the PM of cells in close proximity to the coverslip (up to ~100 nm). PALM acquisition sequence typically took ~2–3 min for two channel imaging at 2000 frame/s. Published and custom algorithms^26, 34, 50–52^ were then applied so that the position map of the identified molecules was further studied, as detailed in the Supplemental Information. For two-color PALM imaging, we used chimeric constructs of molecules with Dronpa or PAmCherry^33^. The fluorescent constructs were imaged with fast alteration of the imaging channel (rate of 52 fps). Photo-activation illumination at 405nm was changed over the imaging sequence of fixed cells to optimize the photo-activation of Dronpa first, and then of PAmCherry at an increased illumination level. Drift compensation and channel registration were performed using dedicated algorithms in the STORM module  of the microscope. Three-dimensional (3D) PALM was conducted using a cylindrical lens that causes astigmatism of the point-spread function (see supplemental information for further details)^52^.

### Data availability

The authors declare that all data that supports the findings of this study are available within the article and its supplementary information files and from the corresponding author upon reasonable request.

## Electronic supplementary material

Supplementary Information
