# Supplementary material for: The L-type Voltage-Gated Calcium Channel co-localizes with Syntaxin 1A in nano-clusters at the plasma membrane
Source: Sci Rep. 2017 Sep 12;7:11350. doi: 10.1038/s41598-017-10588-4 (PMC5595989; doi:10.1038/s41598-017-10588-4)
Supplement: Supplementary file 1 — Supplementary Information [file 41598_2017_10588_MOESM1_ESM.pdf]

Supplementary Information

The L-type Voltage-Gated Calcium Channel co-localizes with Syntaxin 1A in nano-clusters at the plasma membrane

[#≠]Julia Sajman, [#¶]Michael Trus, [≠¶*]Daphne Atlas, [≠*]Eilon Sherman

Key Words: exocytosis, VGCC, Syntaxin 1A;

≠ Racah Institute of Physics
¶ Dept. of Biological Chemistry, Institute of Life Sciences
The Hebrew University of Jerusalem
Jerusalem, 91904 Israel

# Equal contribution

***Correspondence**

Daphne Atlas daphne.atlas@mail.huji.ac.il Phone 972-2-658-5406

Eilon Sherman sherman@phys.huji.ac.il Phone 972-2 658 6878

Data analyses

PALM rendering - PALM images were analysed using the NSTORM module in NIS-Elements (Nikon) or the published ThunderSTORM software [1] to identify peaks and group them into functions that reflect the positions of single molecules [2]. The peak grouping procedure used a distance threshold and a temporal gap to account for possible molecular blinking [3]. The temporal gap was determined for each fluorophore separately in order to minimize over-counting of molecules. Rendering of individual molecules used intensities that correspond to the probability density values of their fitted Gaussians. Considering the maximal probability density values detected in the field set these intensities. Three dimensional PALM was conducted and analysed using the astigmatism method [4]. Calibration was conducting using 100nm Tetraspec fluorescent beads (Invitrogen). Three-dimensional decoding was performed using Nikon NSTORM software.

Second order statistics and pair correlation function

In this study, we used multiple statistical tools to describe the self- and co-clustering of molecules, as detected by PALM. Self-clustering of a single species can be analysed using second order statistics (e.g. univariate pair-correlation function, or PCF) or using a distance-dependent clustering. Co-clustering can be analysed using bivariate second-order statistics (e.g. bivariate PCF, or BPCF). We briefly describe these approaches below, while more details can be found in a recent review [5].

Second order statistics (Fig. S1a) - For two point patterns that represent two different populations, the bivariate PCF, $g_{12}(r)$, is defined as follows [6]:
(1) $g_{12}(r)$=[illegible][The number of points of pattern 2 at distance r from an arbitrary point of pattern 1]
Where [illegible] is the mean density of points of pattern 2 [6]. Following a similar notation to Wiegand and Moloney [6], a bivariate PCF can be calculated for a pixelated image using the following definitions:

(2) $$g_{12}(r) = \frac{A}{n_2} \frac{\frac{1}{n_1}\sum_{k=1}^{n_1} Pnts\left[S_2, R_{1,k}^{w}(r)\right]}{\frac{1}{n_1}\sum_{k=1}^{n_1} Area\left[R_{1,k}^{w}(r)\right]}$$

where, $R_{1,k}^{w}(r)$ is the ring with radius *r* and width *w* centred on the *k*'th point of type *2* (here points of type *2* are simply type *i* molecules, or S_2, as defined above). n_i is the total number of points of type *i* in the study region of area *A*. The operator *Pnts* [S_j,*X*] counts the points of type *j*, namely S_j, in region *X*. The operator *Area* counts the number of cells in the region *X*. The related Wiegand-Moloneyõs $O_{12}(r)$ function is defined by [6]: **.**

For a single population pattern, the univariate PCF is defined as: $g(r) \equiv g_{11}(r)$, in analogy to the $g_{12}(r)$ function.

Molecular clustering (e.g. **Fig. 1c,f**) ï An alternative approach to the second order statistics is to identify clusters of proteins and then study various properties of these clusters. Since all proteins in our sample are equal, we perform non-hierarchical clustering using a nearest-neighbor distance [7], and with an a-priori unknown number of clusters.

Molecular co-clustering analysis (**Fig. 5c,d**) - For the content analysis of co-clusters by two species, we first generated shrunk pictures (each pixel is 100x100 nm containing multiple green and red dots, i.e. molecular positions, in the corresponding area). We defined the clusters' pixels as having at least 50 red dots (i.e., 50/0.01 µm2) and 5 green dots (5/0.01 μm^2). Then we generated a binary picture with pixels filtered according to the above criteria. In this binary picture, we found connected components using the Matlab function 'regionprops'. This step yielded the clusters to be analyzed further for counting their number of incorporated green and red dots. First we got cluster's shrunk picture (now, each pixel is 10x10 nm). Then, we smoothed the clusters (in the picture) with a Gaussian filter (having a standard deviation of 4 pixels) for obtaining a continuous clusters picture. Next, for each color in the smoothed image, we make binary picture using Otsu's method (thresholds are chosen automatically). The resulting binary pictures are used as masks for cluster analyses: Only pixels that have a 'true' value in the mask for both green and red are taken into account and their dots are counted.

Null models ï For interpretation of the statistical analyses above, it is useful to compare them to different models:

The model of **Complete Spatial Randomness (CSR;** e.g. in **Fig 1b,e)** serves to quantify the deviation of a point pattern from randomly distribution, where points are distributed according to the Poisson process. The assumption is that there is no interaction between the points of the pattern and, thus, the points have a constant density as the first order statistics over the study region. The resultant PCF equals 1, i.e. $g(r)$=1, regardless of r.

Considering two different species, two orthogonal processes would result in a **No Interaction (NI) model** (**Fig. S1a**). Here, the model results in flat PCFs with a value of 1, i.e. $g_{12}(r)$ =1, regardless of r. This indicates no interaction (i.e. no spatial correlation) between the species.

The Random Labeling (RL) model (**Fig. S1a**) ï This model serves to investigate whether two species in a joint point pattern significantly interact. In this model, points of pattern 1 (n_1) and points of pattern 2 (n_2) are distributed randomly in (n_1+ n_2) fixed locations. The resultant PCF can then be compared to the original PCF for comparison, as described below.

Multiple Monte-Carlo simulations replicate 19 times the point patterns while randomly re-labelling the points (with the number of points from each species). The bivariate PCF of the original point patter $g_{12}(r)$ is then compare to the bivariate PCFs of the simulations. The lowest and highest $g_{12}(r)$ of 19 different simulations can serve as a 95% confidence interval for the acceptance or rejection of the model as a null hypothesis. Agreement of the data with the RL model indicates homogeneous mixing, and hence strong interaction (in a statistical sense) of the two species under study [8]. Prior knowledge on the physical binding of the two species (e.g. from biochemical assays) can then help to interpret the studied interactions as physical binding events of the species.

Comparing bivariate PCFs of multiple cells

The RL model is individual to each cell and there is no simple way to compare the bivariate PCFs (BPCFs) from multiple cells. Here, we apply two complementary ways to compare and average BPCFs computed for multiple cells: (i) the extent of mixing (EOM), and (ii) the standardized bivariate PCF (SBPCF).

(i) The extent of mixing (EOM; Fig. S1c) - Previously, we have introduced a measure we termed the 'Extent of Mixing' (EOM) [8,9]. As a first step, this measure is computed individually for each cell as follows:

$$(3) \qquad EOM^{i}(r) = \frac{g_{12}^{i}(r) - 1}{g_{12}^{RL,i}(r) - 1}$$

where $g_{12}^{i}(r)$ is the BPCF for cell *i*, and $g_{12}^{RL,i}(r)$ is the average of 19 simulated BPCFs due to the RL model for cell *i*. Next, the EOM can be readily averaged for multiple (N) cells:

$$(4) \qquad EOM(r) = \frac{1}{N}\sum_{i=1}^{N} EOM^{i}(r)$$

The errors are computed as:

$$(5) \qquad SEM(r) = \sqrt{\frac{1}{N^{2}}\sum_{i=1}^{N} EOM(r)^{2}}$$

The EOM typically yields PCF values that range between 0 (for the NI model) and 1 (for the RL model). Thus, it provides an intuitive measure of the extent of interaction between two species. Note that this measure is normalized, and thus, absolute correlation values cannot be compared.

(ii) Standardized bivariate PCF (SBPCF; Fig. S1b) ï To further compare molecular interactions within multiple cells, we took a second approach that preserves the absolute correlation values. Specifically, we define a standardized version of the BPCFs (SBPCF), $\breve{g}_{12}^{i}(r)$, independently for each cell *I*, as follows:

$$(6) \qquad \breve{g}_{12}^{i}(r) = \frac{g_{12}^{i}(r) - \left\langle \tilde{g}_{12}^{i}(r) \right\rangle}{\tilde{\sigma}^{i}(r)}$$

where, $\tilde{g}_{12}^{i}(r)$ is the set of all simulated $g_{12}^{i}(r)$ for cell *i* (i.e. the previous notation $g_{12}^{RL,i}(r)$ in Eqs. 1,2 is now replaced with the more elaborate term $\left\langle \tilde{g}_{12}^{i}(r) \right\rangle$). We further define:

(7) $$\tilde{\sigma}^{i}(r) = \max\left(\left|\left(\tilde{g}_{12}^{i}(r) - \left\langle \tilde{g}_{12}^{i}(r)\right\rangle\right)\right|\right)$$

Here, $2\tilde{\sigma}^{i}(r)$ denotes the 95% confidence interval of the BPCF of cell *i* due to the 19 simulated random sets of the null hypothesis. We then took the average of the SBPCFs and the standard error of the mean (SEM) over multiple *N* cells using the following equations:

(8) $$\left\langle \breve{g}_{12}(r)\right\rangle = \frac{1}{N}\sum_{i=1}^{N}\breve{g}_{12}^{i}(r)$$

(9) $$SEM(r) = \sqrt{\frac{1}{N^{2}}\sum_{i=1}^{N}\breve{g}_{12}^{i}(r)^{2}}$$

Supplementary References

1 Ovesny, M., Krizek, P., Borkovec, J., Svindrych, Z. K. & Hagen, G. M. ThunderSTORM: a comprehensive ImageJ plug-in for PALM and STORM data analysis and super-resolution imaging. *Bioinformatics* **30**, 2389-2390, (2014).

2 Betzig, E., Patterson, G. H., Sougrat, R., Lindwasser, O. W., Olenych, S., Bonifacino, J. S., Davidson, M. W., Lippincott-Schwartz, J. & Hess, H. F. Imaging intracellular fluorescent proteins at nanometer resolution. *Science* **313**, 1642-1645, (2006).

3 Betzig, E., Patterson, G. H., Sougrat, R., Lindwasser, O. W., Olenych, S., Bonifacino, J. S., Davidson, M. W., Lippincott-Schwartz, J. & Hess, H. F. Imaging intracellular fluorescent proteins at nanometer resolution. *Science* **313**, 1642-1645, (2006).

4 Huang, B., Wang, W., Bates, M. & Zhuang, X. Three-dimensional super-resolution imaging by stochastic optical reconstruction microscopy. *Science* **319**, 810-813, (2008).

5 Sherman, E. Resolving protein interactions and organization downstream the T cell antigen receptor using single-molecule localization microscopy: a review. *Methods Appl Fluores* **4**, (2016).

6 Wiegand, T. & Moloney, K. A. Rings, circles, and null-models for point pattern analysis in ecology. *Oikos* **104**, 209-229, (2004).

7 Zhang, J., Leiderman, K., Pfeiffer, J. R., Wilson, B. S., Oliver, J. M. & Steinberg, S. L. Characterizing the topography of membrane receptors and signaling molecules from spatial patterns obtained using nanometer-scale electron-dense probes and electron microscopy. *Micron* **37**, 14-34, (2006).

8 Sherman, E., Barr, V., Manley, S., Patterson, G., Balagopalan, L., Akpan, I., Regan, C. K., Merrill, R. K., Sommers, C. L., Lippincott-Schwartz, J. &

Samelson, L. E. Functional nanoscale organization of signaling molecules downstream of the T cell antigen receptor. *Immunity* **35**, 705-720, (2011).
9 Sherman, E., Barr, V. A. & Samelson, L. E. Resolving multi-molecular protein interactions by photoactivated localization microscopy. *Methods* **59**, 261-269, (2013).

Supplementary Figure Legends

Figure S1. Second-order statistical analyses of molecular interactions for single and multiple cells

(a) Bivariate pair correlation function (BPCF) of two molecular species in single cells. The BPCF (bold blue line) is compared to a model of random labeling (solid black lines) or to a model of no-interaction (dashed green line) (**b**) The average of the standardized BPCF over multiple cells (solid blue line). The SBPCF is compared to a model of random labeling at ±1(solid black lines) or to a model of no-interaction (bold green line). Error bars are SEM (**c**) The of the extent of mixing (EOM) (solid blue line) for multiple cells. The EOM is compared to a model of random labeling at +1(dashed black line) or to a model of no-interaction at 0 (dashed green line). Error bars are SEM. See further details in the Analyses section of the Supplementary information.

Figure S2. Cluster size analyses of Sx1A and Cav1.2

(a) A histogram of the area of Sx1A clusters. The histogram of the clusters (red line) is compared to the histogram of clusters found in simulated Poisson-distributed random sets (dashed blue line) (**b**) The dependence of the average size in copy number of Sx1A clusters on the number of detected Sx1A molecules at the PM of individual cells by PALM (red diamonds). The data is compared to the average cluster size found in simulated Poisson-distributed random sets (blue dots) (**c**) The dependence of the average area of Sx1A clusters on the number of detected Sx1A molecules at the PM of individual cells by PALM (red diamonds). The data is compared to the average cluster size found in simulated Poisson-distributed random sets (blue dots) **(d)** A histogram of the area of Cav1.2 clusters. The histogram of the clusters (green line) is compared to the histogram of clusters found in simulated Poisson-distributed random sets (dashed blue line) (**e**) The dependence of the average

size in copy number of Cav1.2 clusters on the number of detected Cav1.2 molecules at the PM of individual cells by PALM (green diamonds). The data is compared to the average cluster size found in simulated Poisson-distributed random sets (blue dots) (**f**) The dependence of the average area of Sx1A clusters on the number of detected Cav1.2 molecules at the PM of individual cells by PALM (green diamonds). The data is compared to the average cluster size found in simulated Poisson-distributed random sets (blue dots). Black trend lines in panels b,c,e,f are linear fits to the experimental data.

Figure S3. Effect of cytosolic domain of Sx1A on interaction with Cav1.2

The extent of mixing is shown for the conditions presented in **Fig. 2b,d**; Fig. 3b,d,f; **Fig. 4b**, **Fig. 5b,d,f**; **Fig. 6b,d.** It is derived from the standardized bivariate PCSs of the Cav1.2-Dronpa and the PAmCherry-Sx1A proteins at the specified panels (see Methods and Fig. S1 for further details); [1 =complete mixing; 0 = no interaction]

Figure S4. Self-clustering of Sx2 and Sx1A mutants

(**a**) A PALM image of a representative HEK293 cell expressing mutant Sx1A (VV)-PAmCherry. Zoom image on right shows self-clusters. Bars ï 5µm (*left*) and 0.6 µm *(right*) (**b**) The average Pair correlation function of Sx1A$^{CC/VV}$ in multiple cells (N=46). Errors are SEM (**c**) A Cumulative size-distribution curves of the Sx1A$^{CC/VV}$ self-clusters (N=46 cells) (**d**) A PALM image of a representative HEK293 cells expressing Sx2-PAmCherry. Zoom image on right shows self-clusters. Bars ï 5 mm (l*eft*) and 0.6 µm (*right*). (**e**) The average Pair correlation function of Sx2 in multiple cells (N=26). Errors are SEM (**f**) A Cumulative size-distribution curve of the Sx2 self-clusters. (N=26 cells) (**g**) A PALM image of a representative HEK293 cells expressing Sx2/Sx1A-PAmCherry. Zoom image on right shows self-clusters. Bars ï 5 µm (*left*) and 0.6 µm (*right*) (**h**) The average Pair correlation function of Sx2/Sx1A in multiple cells (N=17). Errors are SEM (**i**) A Cumulative size-distribution curve of the /Sx1ASx2 self-clusters. (N=17 cells) (**j**) A PALM image of a representative HEK293 cells expressing PAmCherry-Sx1A^{C145A}. Zoom image on right shows self-clusters. Bars ï 5 µm (*left*) and 0.6 µm (*right*) (**k**) The average Pair correlation function of Sx1A^{C145A} in multiple cells (N=34). Errors are SEM (**l**) A Cumulative size-distribution curve of the Sx1A^{C145A} self-clusters (N=34 cells).

Figure S5. Sx1A form clusters with $\alpha_1$1.2 as opposed to Sx2, Syx2/Syx1A chimera, or Sx1A^{C145A}

Figure S6. Mutation or oxidation of Cys271 and Cys271 in Sx1A trans-membrane domain affects clustering ratio with the channel

Figure S1

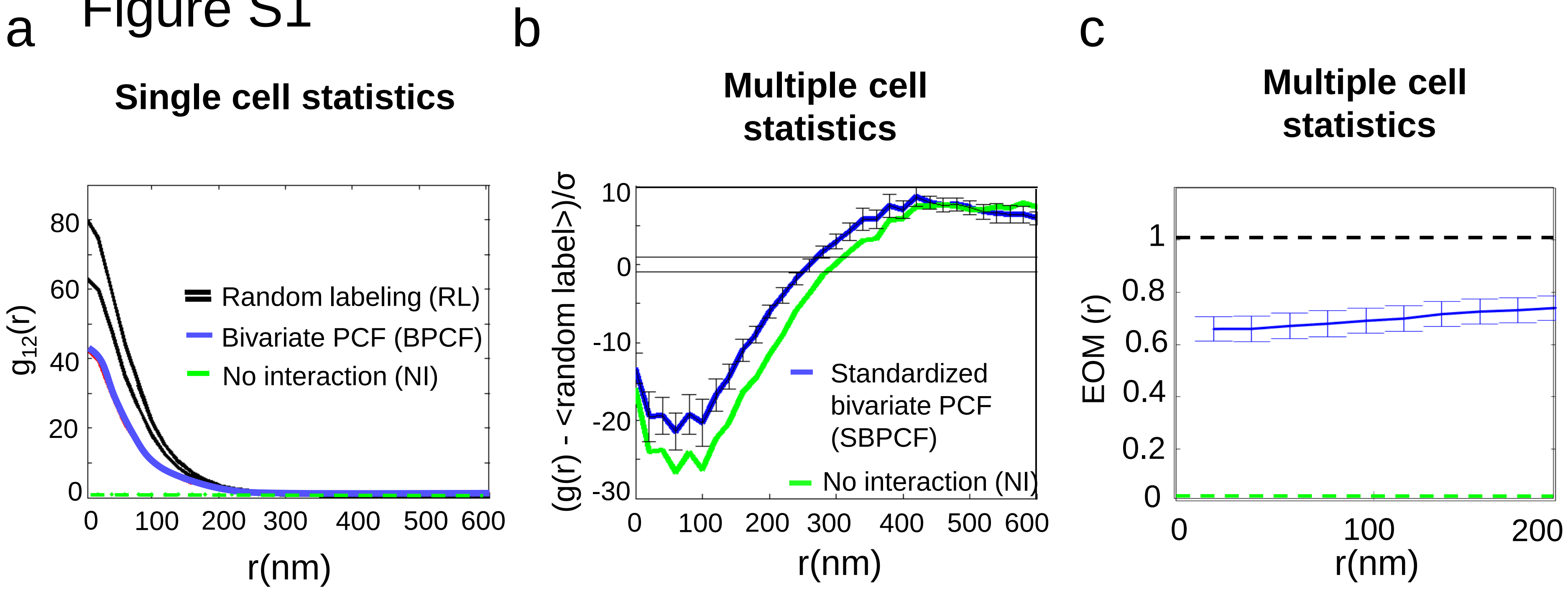

— $\langle g_{12}^{RL}\rangle = \frac{[(E^{+}(r))+(E^{-}(r))]}{2}$

$\sigma(r) = \frac{[(E^{+}(r))-(E^{-}(r))]}{2}$

— SBPCF $= \frac{g_{12}(r)-\langle g_{12}^{RL}\rangle}{\sigma(r)}$

— $\frac{1-\langle g_{12}^{RL}\rangle}{\sigma(r)}$

— Extent of mixing (EOM)

$= \frac{g_{12}(r)-1}{\langle g_{12}^{RL}(r)\rangle-1}$

Figure S2

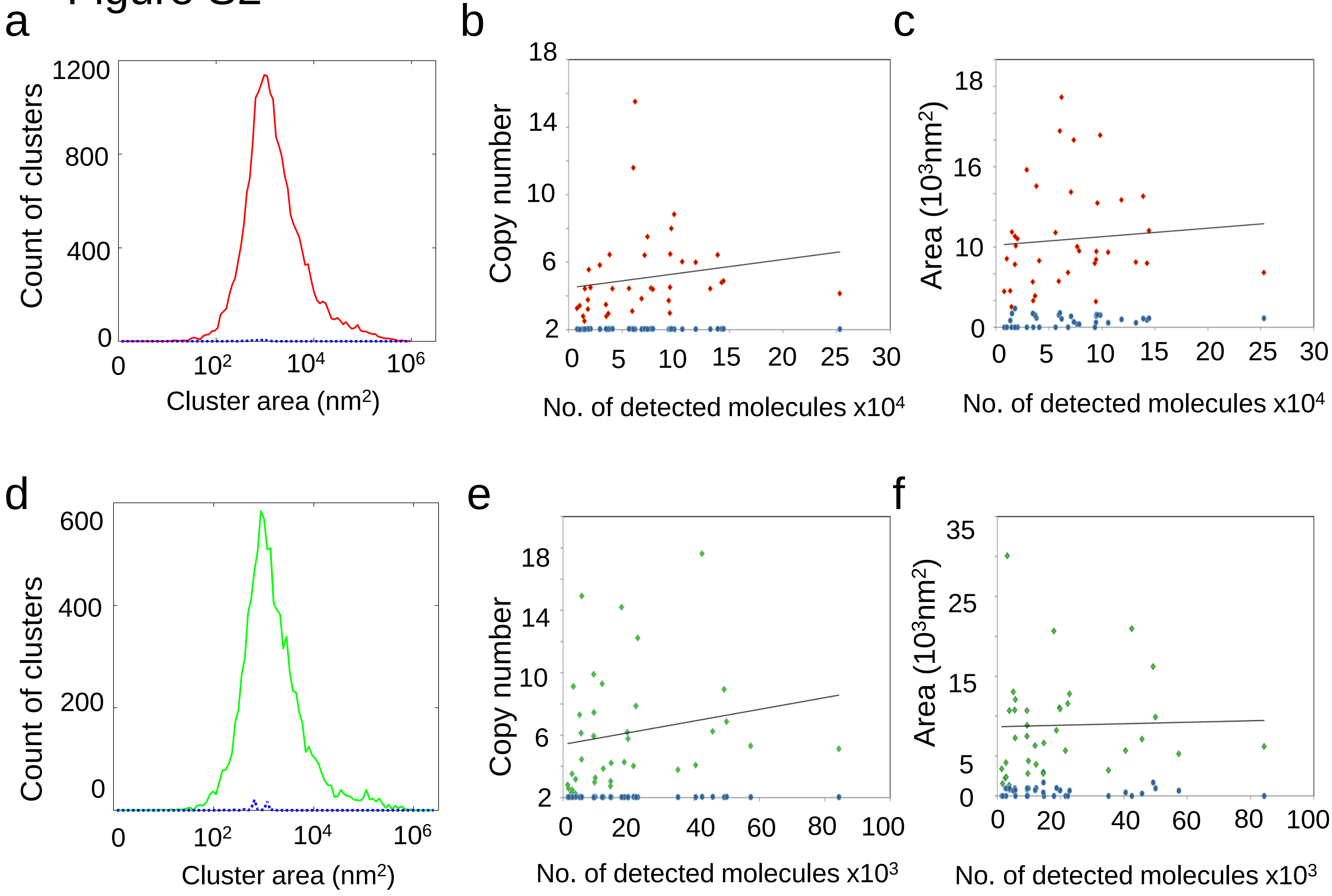

a
Count of clusters
1200
800
400
0
0
10²
10⁴
10⁶
Cluster area (nm²)
b
Copy number
18
14
10
6
2
0
5
10
15
20
25
30
No. of detected molecules x10⁴
c
Area (10³nm²)
18
16
10
0
0
5
10
15
20
25
30
No. of detected molecules x10⁴
d
Count of clusters
600
400
200
0
0
10²
10⁴
10⁶
Cluster area (nm²)
e
Copy number
18
14
10
6
2
0
20
40
60
80
100
No. of detected molecules x10³
f
Area (10³nm²)
35
25
15
5
0
0
20
40
60
80
100
No. of detected molecules x10³

Figure S3

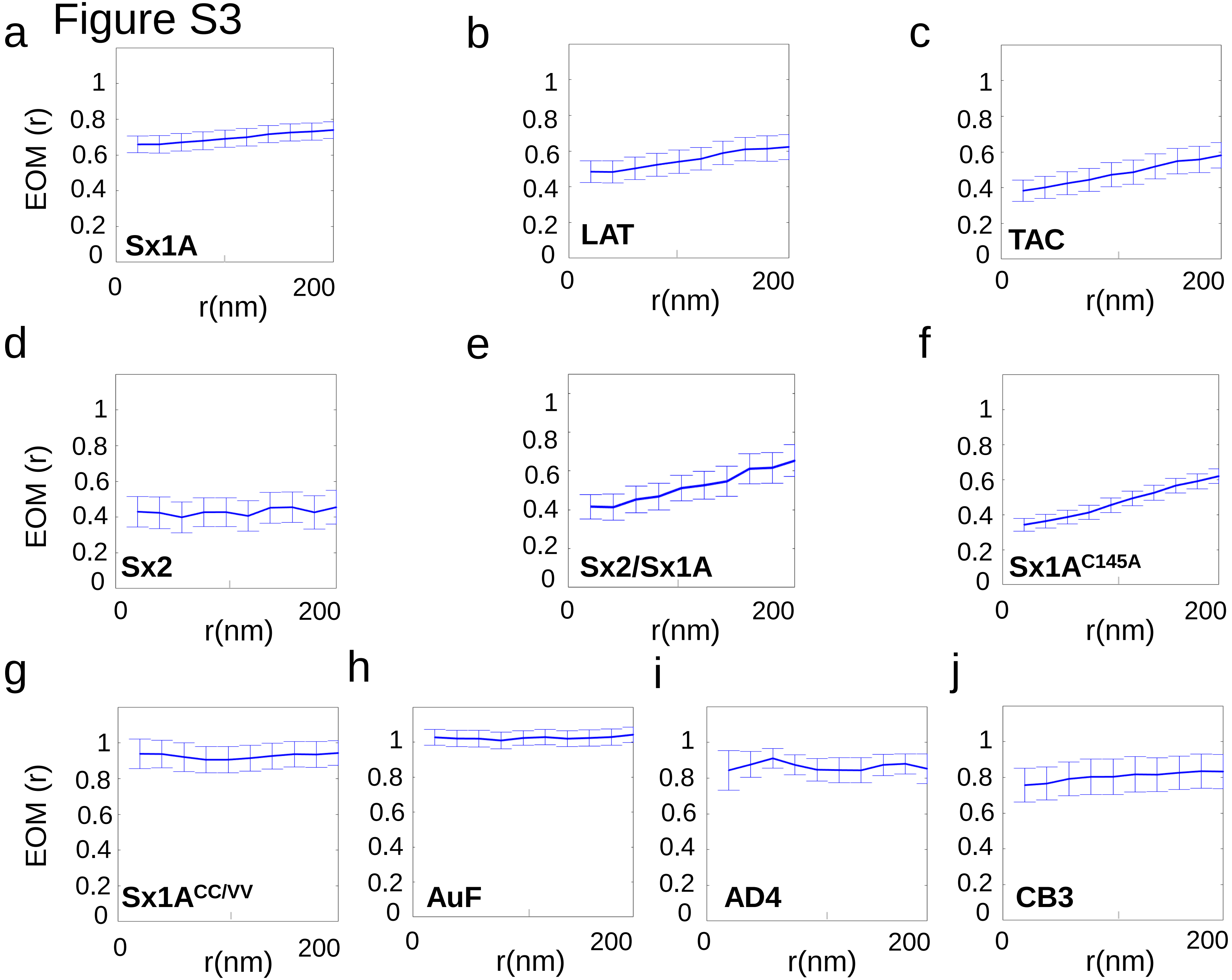
a
EOM (r)
1
0.8
0.6
0.4
0.2
0
Sx1A
0
200
r(nm)
b
LAT
c
TAC
d
Sx2
e
Sx2/Sx1A
f
Sx1A^{C145A}
g
Sx1A$^{CC/VV}$
h
AuF
i
AD4
j
CB3

Figure S4

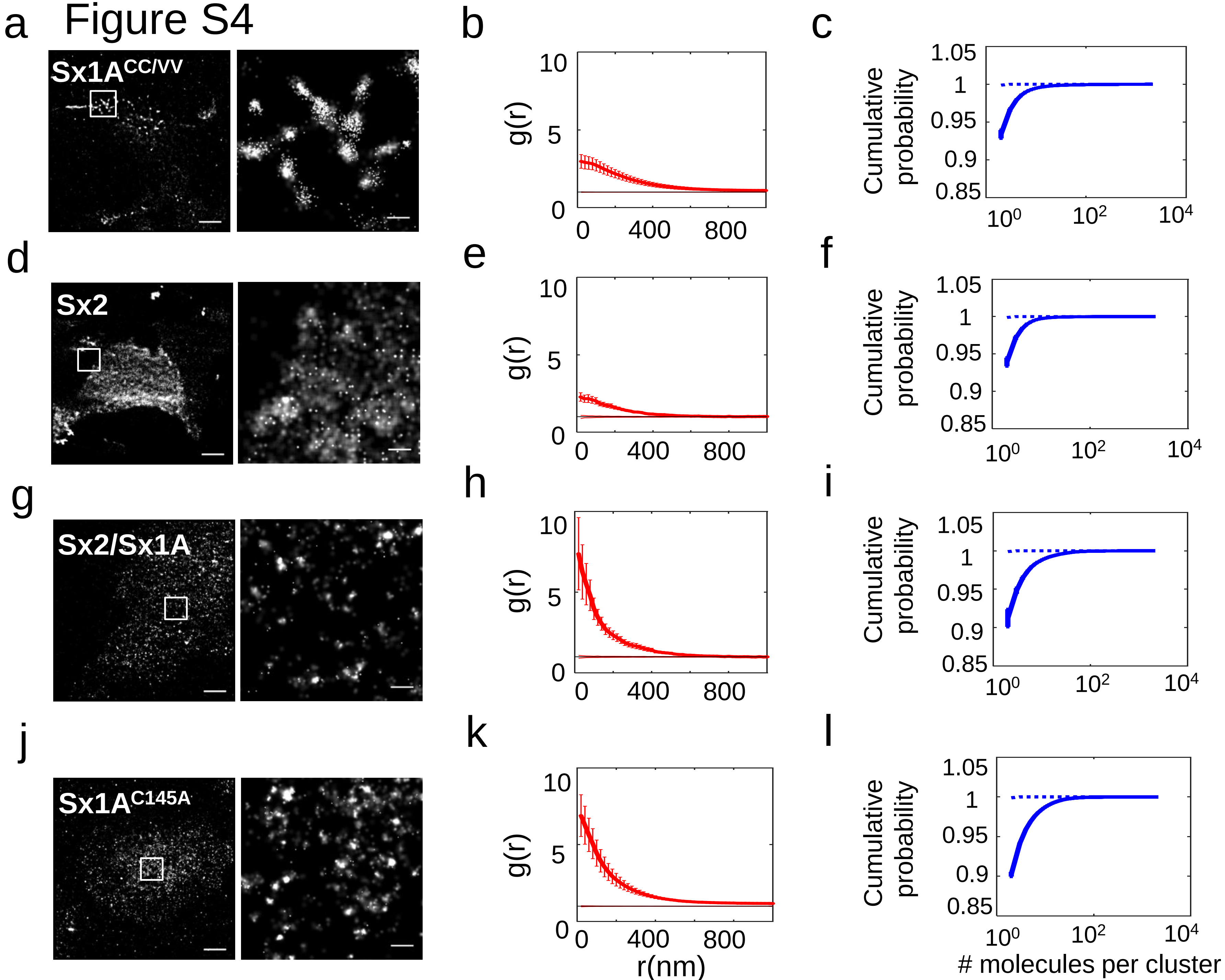
a
b
c
Sx1A$^{CC/VV}$
d
e
f
Sx2
g
h
i
Sx2/Sx1A
j
k
l
Sx1A^{C145A}
g(r)
r(nm)
Cumulative probability
molecules per cluster
10
5
0
0
400
800
1.05
1
0.95
0.9
0.85
10^0
10^2
10^4

Figure S5

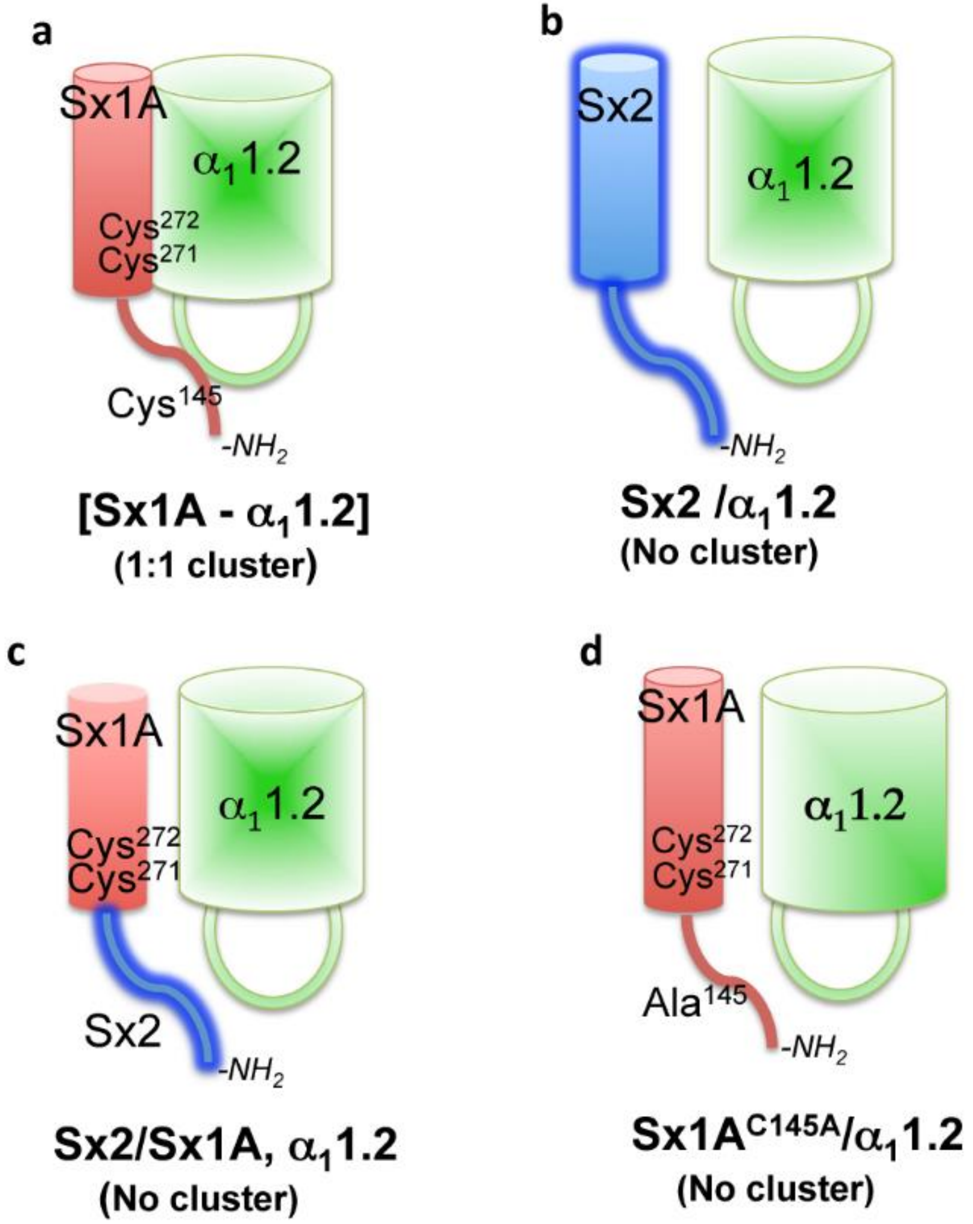

a
Sx1A
α1 1.2
Cys272
Cys271
Cys145
-NH2
[Sx1A - α1 1.2]
(1:1 cluster)
b
Sx2
α1 1.2
-NH2
Sx2 /α1 1.2
(No cluster)
c
Sx1A
α1 1.2
Cys272
Cys271
Sx2
-NH2
Sx2/Sx1A, α1 1.2
(No cluster)
d
Sx1A
α1 1.2
Cys272
Cys271
Ala145
-NH2
Sx1AC145A/α1 1.2
(No cluster)

Figure S6

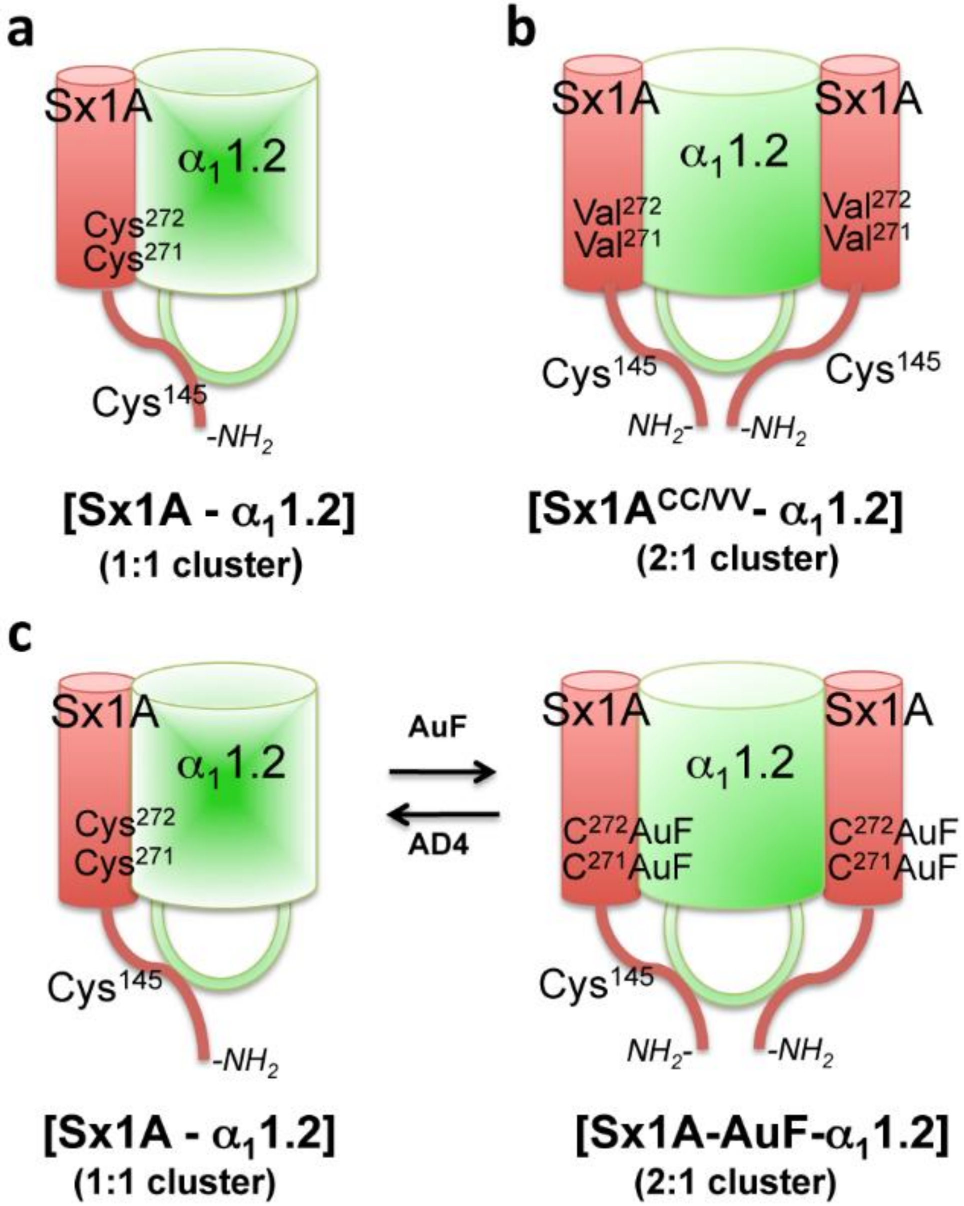
a
Sx1A
α1 1.2
Cys272
Cys271
Cys145
-NH2
[Sx1A - α1 1.2]
(1:1 cluster)
b
Sx1A
Sx1A
α1 1.2
Val272
Val271
Val272
Val271
Cys145
Cys145
NH2-
-NH2
[Sx1A CC/VV- α1 1.2]
(2:1 cluster)
c
Sx1A
α1 1.2
Cys272
Cys271
Cys145
-NH2
AuF
AD4
Sx1A
Sx1A
α1 1.2
C272AuF
C271AuF
C272AuF
C271AuF
Cys145
NH2-
-NH2
[Sx1A - α1 1.2]
(1:1 cluster)
[Sx1A-AuF-α1 1.2]
(2:1 cluster)
